# METER (Mental health emergency response) program: Findings of psychological impact status and factors associated with depression, anxiety and stress among healthcare workers in public hospital in Malaysia during the COVID-19 pandemic

**DOI:** 10.1371/journal.pgph.0001823

**Published:** 2023-04-14

**Authors:** Nor Asiah Muhamad, Natasha Subhas, Normi Mustapha, Norni Abdullah, Muhammad Arif Muhamad Rasat, Rimah Melati AB Ghani, Fatin Athira Tahir, Anne Nik Ismaliza Ishak, Vevehkanandar Sivasubramaniam, Alinazarine Hassan, William Wei Liang Goh, Kok Liang Teng, Ainul Izzah Abdul Manan, Rosmawati Mokhtar, Amrit Kaur Baljit Singh, Kher Shean Ng

**Affiliations:** 1 Sector for Evidence Based Healthcare, National Institutes of Health, Ministry of Health, Shah Alam, Selangor, Malaysia; 2 Department of Psychiatry and Mental Health, Hospital Tengku Ampuan Rahimah, Ministry of Health, Klang, Malaysia; 3 Faculty of Science & Technology, Open University Malaysia, Petaling Jaya, Malaysia; 4 Institute for Public Health, National Institutes of Health, Ministry of Health, Shah Alam, Malaysia; University of the Witwatersrand, SOUTH AFRICA

## Abstract

**Introduction:**

The COVID-19 pandemic has become the greatest challenge of the new millennium. Most healthcare workers (HCWs) experienced unprecedented levels of workload since the pandemic. This study aims to identify the prevalence and factors of depression, anxiety and stress among HCWs in Malaysian healthcare facilities in the midst of the pandemic due to the SARs-CoV-2.

**Methods:**

An emergency response programme on mental health was conducted from June to September 2020. A standardized data collection form was distributed among the HCWs in the government hospital in Klang Valley. The form contained basic demographic information and the self-reported Malay version of the Depression, Anxiety and Stress scale (BM DASS-21).

**Results:**

Of the1,300 staff who attended the Mental Health and Psychosocial Support in Covid-19 (MHPSS COVID-19) programme, 996 staff (21.6% male, 78.4% female) completed the online survey (response rate: 76.6%). Result showed that staff aged above 40 years old were almost two times more likely to have anxiety (AOR = 1.632; 95% CI = 1.141–2.334, p:0.007) and depression (AOR = 1.637; 95% CI = 1.1.06–2.423, p:0.014) as compared to staff who were less than 40 years old. Those who had direct involvement with COVID-19 patients were likely to suffer stress (AOR = 0.596; 95% CI = 0.418–0.849, p:0.004), anxiety (AOR = 0.706; 95% Ci = 0.503–0.990, p:0.044) and depression (AOR = 0.630; 95% Ci = 0.427–0.928, p:0.019). HCWs with stress (AOR = 0.638; 95% CI of 0.476–0.856, p = 0.003), anxiety (AOR = 0.720; 95% CI 0.542–0.958, p = 0.024) and depression (AOR = 0.657; 95% CI 0.480–0.901, p = 0.009) showed less confidence to treat critically ill patients and need psychological help during outbreak.

**Conclusion:**

This study showed the importance of psychosocial support to reduce psychological distress among HCWs when working or coping during the COVID-19 pandemic or outbreak.

## 1. Introduction

The coronavirus disease 2019 (COVID-19) first emerged in December 2019 in Wuhan City, China following reports of several pneumonia cases with unknown aetiology agent [1]. The aetiological agent was soon identified as severe acute respiratory syndrome coronavirus 2 (SARS-CoV-2) [2, 3]. COVID-19 was considered as a Public Health Emergency of International Concern (PHEIC) by World Health Organization (WHO) due to its rapid spread and declared a pandemic on 12 March [4–6].

Malaysia and other Southeast Asian countries were among the first few countries to report incidences of the virus outside China [7, 8]. The first confirmed case of COVID-19 in Malaysia was detected among three Chinese nationals on 25 January 2020 who previously had close contact with people infected with COVID-19 in Singapore [7, 8]. However, following the rapid increase in active COVID-19 cases, the Malaysian Government ordered the Movement Control Order (MCO) beginning on 18 March 2020 [9]. The MCO was enforced as a mitigation effort to reduce community spread and decrease the overburdening of the country’s health system [10].

Psy**c**hological distress during a pandemic is common, be it to the general public or health care workers. Various studies have reported the consequences of the first massive disease outbreak of the 21st century, severe acute respiratory syndrome (SARS) [11]. These studies found that in the immediate aftermath of the SARS outbreak, psychiatric problems commonly diagnosed were adjustment reactions, stress and increased anxiety levels [11–16]. A study conducted during the SARS outbreak in a large teaching hospital in Toronto reported almost two-thirds of healthcare workers (HCWs) surveyed experienced high levels of psychiatric distress [17]. Working during or immediately after an outbreak of an infectious disease or pandemic which may affect the healthcare system, will have a negative impact on the mental health and well-being of the HCWs.

Various factors will affect the mental health and well-being of the HCWs during a pandemic such as concern about exposure to the virus; personal and family needs and responsibilities; managing a different workload; lack of access to necessary tools and equipment (including personal protection equipment, PPE), learning new technical skills and adapting to a different work [18]. Six months’ post-discharge, major depression (23.6%), adjustment disorder (8.1%) and post-traumatic stress disorder (PTSD) (7.3%) were common psychopathologies seen in SARS patients [19]. A study by Mak et al., (2009) reported high incidences of mental health disorders at 58.9% following the SARS outbreak [11].

As mentioned above, common psychological sequelae seen during and after a pandemic are stress, depression and anxiety among HCWs as well as the general population [11, 20, 21]. Recent studies like Wang C et al. [22] and Qiu J et al., [23] found that their community suffered from severe psychological distress (stress, anxiety and depression) during the COVID-19 pandemic. From experience, stress, depression and anxiety are not only common amongst the general population, the prevalence and risk are significantly higher for the high-risks populations such as healthcare workers [24, 25].

Healthcare workers not only engage in the treatment of infected patients, but are also at risk of being infected themselves. Apart from the risk of being of infected, they face additional stressors, such as the fear of transmitting COVID-19 to family members, being stigmatized or rejected by others based on their occupation, and working under highly stressful conditions [26]. Over time, the increasing COVID-19 cases with associated deaths, a heavy workload for long periods and limited availability of personnel protective equipment could further cause physical and emotional burnout in these healthcare workers [26]. There are several studies conducted on healthcare workers’ mental health during the COVID-19 pandemic. Pappa S et al. [27] reported that one in every five healthcare workers suffers from depression, anxiety or both. Another review concluded that healthcare workers reported more anxiety, depression and sleep problems [28]. Badahdah A et al. [29]’s study in Oman found that there was a high prevalence of stress, anxiety and poor psychological well-being amongst young health care workers during the COVID-19 pandemic. Studies from China found that frontline HCWs in direct contact with COVID-19 patients suffered stress, anxiety and insomnia and higher levels of psychiatric symptoms compared to those with indirect contact [30, 31]. A large, retrospective cohort study involving 62,354 participants in the United States by Taquet et al., 2021 reported survivors of COVID-19 appear to be at increased risk of psychiatric sequelae, and a psychiatric diagnosis might be an independent risk factor for COVID-19 [32]. Therefore, it is pertinent to place importance on early detection and management of psychological sequelae in case of future pandemic outbreaks to avoid a ‘Mental Health Catastrophe’ [11, 31].

COVID-19 is itself a huge stressor, as there were no existing medicine or vaccines during the start of the pandemic [33]. Stress can be defined as a ‘state of disharmony and is neutralized by a complicated variety of physiologic and behavioural responses that aim to maintain/re-establish the threatened homeostasis (adaptive stress response)’ [34, 35]. Stress has a wide spectrum of symptoms ranging from emotional to physical disorders like sadness, anxiety, palpitations, and gastrointestinal distress. The higher the stress, the more its symptoms like frequent headaches, fatigue, neck/back pain, excessive worries, muscle tension and feeling overwhelmed [34]. Many people experience poor concentration, forgetfulness and low energy as a stress response to the pandemic [34]. Depression is a common but complex disorder with a range of unique symptomology that includes persistent low mood, anhedonia, poor concentration, sleep disturbances, fatigue and in more severe forms lead to impairment in function and suicidal ideations [36, 37]. Anxiety disorder is an anxiety disorder characterised by excessive overthinking and worry, feeling at the edge, muscle tension, poor concentration associated with symptoms of hypervigilance, and other somatic symptoms of anxiety [36, 38]. Another common anxiety disorder, panic disorder, is characterised as sudden, sometimes unexpected paroxysmal bursts of severe anxiety usually associated with several physical symptoms (autonomic, otoneurological, cardiorespiratory or gastrointestinal) which can be disabling and affect a person’s function [36, 39]. The Depressive, Anxiety and Stress Scale (DASS) was developed specifically to measure depression, anxiety and stress levels concurrently [40, 41]. Each of the three subscales of DASS is intercorrelated with another [40, 41]. During the COVID‐19 coronavirus pandemic WHO warned about the potential negative impact of the crisis on the psychological and mental well‐being of the population particularly health and social care professionals [42]. Thus, evaluating how individuals perceive stressful situations in their lives is critical for the quantification of psychological stress among HCWs.

An increase in psychological distress, anxiety and depression showed a need for a programme to support psychologically the HCWS and other professionals [18, 23, 29]. Developing and promoting resilience should protect people from stress and psychopathological symptoms during the COVID-19 outbreak [37]. Even though perseverance and resilience are associated with mental health outcomes, no data are available for HCWs in Malaysia. Therefore, this study aimed to determine the prevalence of depression, anxiety, and stress among HCWs in public hospitals in Malaysia. This study hypothesised a relevant prevalence rate for moderate to severe psychological distress among HCWs. The negative impact on health and social care professions may result in effects at multiple levels, from the individual worker to the entire health and social care system at the macro level. The finding from this study is useful to assess the health and well‐being of HCWs during the COVID-19 pandemic and provide psychological support to HCWs for the sustainability and functionality of the workforce and healthcare system.

## 2. Methods

### Ethics statement

This study approval was obtained from the National Medical Ethics Committee with NMRR ID-21-02186-QTG and Medical Research Ethics Committee at the National Institutes of Health, Malaysia. Subjects of this study consented to participation in this study before data collection.

### Study design, participant and setting

During the movement control order, a serial programme on Mental Health and Psychosocial Support in Covid-19 (MHPSS COVID-19) was activated in all tertiary hospitals in Klang Valley, Malaysia throughout 2020. The serial programme on MHPSS COVID-19 was planned for all HCWs in various clinical fields managing COVID-19 patients at Hospital. The serial programme on MHPSS COVID-19 consists of education on stress management, relaxation technique, and hotline access for early psychological and emotional support. The MHPSS COVID-19 providers consisted of psychiatrists, medical officers, clinical psychologists and counsellors who were tasked to assess the mental health status of all the HCWs of their hospitals.

A cross-sectional questionnaire-based survey was conducted throughout 2020. HCWs working at the government hospital within the Ministry of Health, Malaysia who attended the MHPSSS COVID -19 were eligible to participate in the survey. Exclusion criteria included those under 18 years of age, non-Malay speakers, and non-HCWs. The study participants were recruited during this programme. Those who agreed to participate were subsequently asked to respond to the Malay version of the Depression, Anxiety and Stress scale (Malay Version of DASS-21). The assessment was conducted through a google form and subsequently, Psychological First Aid (PFA) and Virtual Psychological Intervention (Please see the description in the sub-section) were provided to the HCWs who had psychological distress.

### Data collection

Upon recruitment, a Psychological First Aid (PFA) form was distributed online as a first aid screening which was sent out via QR code and electronic messaging system to all participants in the programme. Subsequently the Virtual Psychological Intervention were provided to the participants who had psychological distress beside the Psychological First Aid (PFA). Participants who agreed to participate were subsequently asked to respond to the Malay Version of DASS-21 via a google form. We adopted the modified version of the Depression, Anxiety, and Stress Scale−21 (Malay Version of DASS-21), which is a reliable and valid self-administered instrument to screen for these psychological disorders. The Malay version of the DASS 21 item is a modified version of the original 42 items (DASS 42) and is a self-reported instrument requiring no special skills to administer. The malay version of the DASS-21 has good psychometric properties and is culturally suitable to be used for the Malaysian general population [40, 41].

### Psychological first aid screening application and reporting

**Fig 1:**
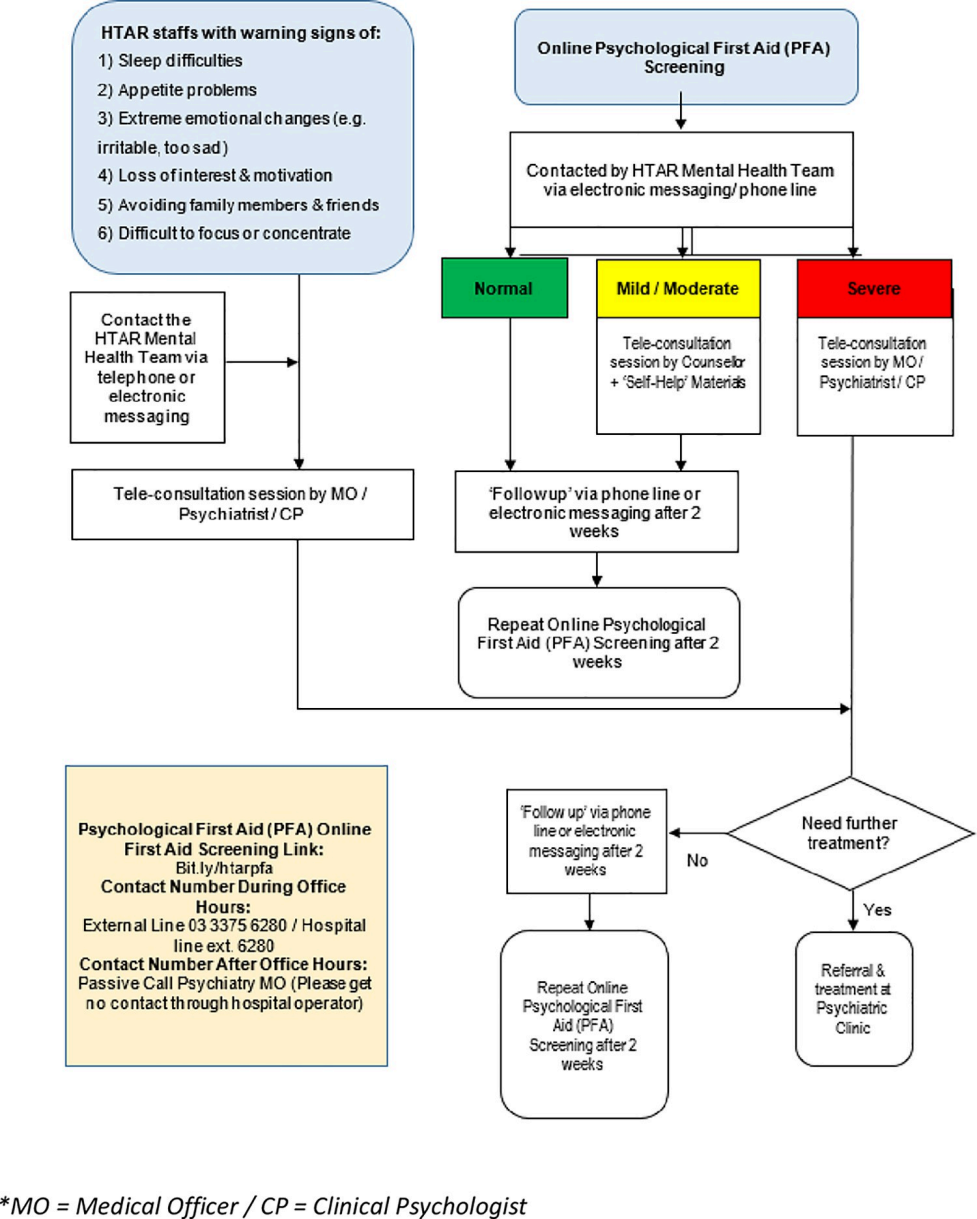
Flow chart of Psychological First Aid (PFA), Mental Health Team, Hospital Tengku Ampuan Rahimah (HTAR). *MO = Medical Officer / CP = Clinical Psychologist.

All participants in the programme received a Psychological First Aid (PFA) screening form online via QR code scanning or an electronic messaging system. The PFA form contained two main sections. The first section contains information on socio-demographic such as gender, current age, ethnicity, nationality, profession, and involvement with COVID-19 patients. The second section contains information on the Malay version of the Depression, Anxiety and Stress scale (BM DASS-21). The DASS-21 scoring was coded as normal, mild, moderate, and severe. All HCWs received their results upon completion of the PFA screening. Any HCWs with a normal score received a phone call or electronic message after two weeks. For HCWs who scored ‘mild/moderate’ were requested to attend a teleconsultation session by the counsellor with ‘Self-help’ materials. Other HCWs with a ‘severe’ score were asked to attend a teleconsultation session by the clinical psychologist or medical officer or psychiatrist (Fig 1).

### Statistical analysis

Univariate, bivariate, and multivariate statistical analyses were conducted. Frequency and percentages were used to describe characteristics and estimate prevalence rates of depression, anxiety, and stress among participants. A bivariate analysis was conducted using Pearson’s chi-square test to explore the association between sociodemographic traits and psychological characteristics and support with each DASS subscale. Variables that were significantly (p-value is considered to be less than 0.05) associated with the outcomes were further analysed by entering the adjusted multivariate model. Multivariate logistic regression analysis determined the factors associated with each outcome (i.e., depression, anxiety, and stress). Adjusted odds ratios with 95% confidence intervals (CIs) and p-values were calculated to determine the strength and significance of the association. All statistical analyses were performed using SPSS, version 22.0 (SPSS Inc., Chicago, Ill., USA).

## 3. Results

### Participants’ characteristics

A total of 1300 staff participated in the MHPSS COVID-19 programme. Of these, 996 completed the online survey which gave a response rate of 76.6%. Of the total participants, 78.4% (781) were female, 80.2% (799) were below 40 years of age and 68.7% (684) were support staff (Table 1). Table 2 showed the distribution of depression, anxiety and stress among health workers.

**Table 1:** Distribution of sociodemographic characteristics of the healthcare workers (N = 996).

Characteristic/ Variable	frequency (%)
Gender	
Male	215 (21.6)
Female	781 (78.4)
Job Category	
Support Staff	684 (68.7)
Technical Staff	312 (31.3)
Age Group	
< 40 years old	799 (80.2)
≥ 40 years old	197 (19.8)

**Table 2:** Distribution of depression, anxiety and stress among healthcare workers (N = 996).

Characteristic/ Variable	frequency (%)
Depression Category	
Normal	775 (77.8)
Mild	104 (10.4)
Moderate	71 (7.1)
Severe	46 (4.6)
Depression	
No	775 (77.8)
Yes	221 (22.2)
Anxiety Category	
Normal	716 (71.9)
Mild	71 (7.1)
Moderate	125 (12.6)
Severe	84 (8.4)
Anxiety	
No	716 (71.9)
Yes	280 (28.1)
Stress Category	
Normal	727 (73.0)
Mild	186 (18.7)
Moderate	61 (6.1)
Severe	22 (2.2)
Stress	
No	727 (73.0)
Yes	269 (27.0)

With regards to the relaxation tips being provided by the hospital’s psychological response team in the google form, 955 staff (95.9%) found it useful. The survey revealed 892 staff (89.6%) were worried about contracting COVID-19 and 942 (94.6%) were worried they may spread it to their family members. Although 903 staff (90.7%) were worried about the shortage of Personal protective equipment, 514 staff (51.6%) were quite confident about treating critical patients.

A total of 493 (49.5%) of the staff felt that adequate working hours during the pandemic should be between 6 to 8 hours, followed by 4 to 6 hours (28.8% or 267) and 8 to 10 hours (124 or 12.4%). During the pandemic, 733 (73.6%) of the staff felt that they had time to rest physically and mentally. A total of 866 staff (86.9%) were satisfied with how the hospital was supporting them during the pandemic and 995 (95.6%) of the staff declined psychological intervention provided by the hospital. The staff were asked to fill up the online DASS-21 scoring, a tool used to measure psychological distress (Table 3).

**Table 3:** Distribution of psychological characteristics among healthcare workers (N = 996).

Characteristic/ Variable	frequency (%)
Involvement with Covid 19 patients	
No	320 (32.1)
Direct	275 (27.6)
Indirect	401 (40.3)
Do you think these tips are useful to you and can be practiced in the current situation?	
No	41 (4.1)
Yes	955 (95.9)
Are you worried that you will be infected with Covid-19?	
No	104 (10.4)
Yes	892 (89.6)
Are you afraid that you could infect your family?	
No	54 (5.4)
Yes	942 (94.6)
Are you worried about the lack of PPE (Personal protective equipment)?	
No	93 (9.3)
Yes	903 (90.7)
Do you feel less confident to treat critically ill patients?	
No	514 (51.6)
Yes	482 (48.4)
4 to 6 hours	267 (26.8)
In your opinion, in one day, what is the optimal time to work in the current situation?	
Less than 4 hours	79 (7.9)
6 to 8 hours	493 (49.5)
8 to 10 hours	124 (12.4)
More than 10 hours	33 (3.3)
Do you have the opportunity of time to rest (physically or mentally) during the handling of this COVID-19 outbreak?	
No	263 (26.4)
Yes	733 (73.6)
Are you satisfied with the hospital’s support for all of you during the handling of this COVID-19 outbreak?	
No	130 (13.1)
Yes	866 (86.9)
Do you need psychological help from the Counseling Unit / Department of Psychiatry and Mental Health HTAR?	
No	955 (95.9)
Yes	41 (4.1)

### Depression

With regards to depression, this study (Table 2) showed that about 77.8% (775) were normal, 10.4% (104) were mild, 7.1% (71) were moderate, and 4.6% (46) were severe. Therefore, 77.8% (775) were normal and 22.2% (221) had depression. Staff aged below 40 years old were twice more likely to have depression as compared to those aged 40 and above (AOR = 1.637; 95% CI: 1.106–2.423, p = 0.014) (Table 4). Staff with no involvement with COVID-19 patients are less likely to have depression (p = 0.011) as compared to those who have direct involvement with COVID-19 patients (AOR = 0.630; 95% CI:0.427–0.928, p = 0.019). Staff who have no time to rest during the COVID-19 pandemic are twice more likely to have depression as compared to those who have an adequate amount of rest (AOR = 1.546; 95% CI: 1.106–2.160, p = 0.011). Staff who were not satisfied with the hospital’s support were 2.5 times more likely to have depression as compared to those who were satisfied with the hospital during the COVID-19 pandemic (AOR = 2.483; 95% CI: 1.649–3.739, p<0.05). Staff who did not require psychological intervention provided by the hospital were less likely to have depression as compared to those who need of psychological support (AOR = 0.172; 95% CI: 0.112–0.264, p<0.05).

**Table 4:** Multiple logistic regression between sociodemographic, psychological characteristics and support with depression, anxiety and stress among healthcare workers (N = 996).

Variables		Depression						Anxiety						Stress					
		Outcome		Adjusted OR	95% CI		*p* value	Outcome		Adjusted OR	95% CI		*p* value	Outcome		Adjusted OR	95% CI		*p* value
		Normal	Depression		Lower	Upper		Normal	Anxiety		Lower	Upper		Normal	Stress		Lower	Upper	
Age	< 40 years	611	188					560	239					575	224				
	≥ 40 years	164	33	1.637	1.106	2.423	**0.014**	156	41	1.632	1.141	2.334	**0.007**	152	45	1.404	0.983	2.007	0.062
Involvement with COVID-19 patients	No	270	50				0.011	244	76					257	63				0.003
	Direct	202	73	0.630	0.427	0.928	**0.019**	200	75	0.706	0.503	0.990	**0.044**	190	85	0.596	0.418	0.849	**0.004**
	Indirect	303	98	1.181	0.820	1.703	0.372	272	129	0.812	0.574	1.149	0.240	280	121	1.129	0.803	1.589	0.484
Are you worried that you will be infected with COVID-19?	No	78	26					72	32					70	34				
	Yes	697	195	1.295	0.780	2.148	0.317	644	248	1.249	0.784	1.990	0.349	657	235	1.963	1.173	3.284	**0.010**
Are you afraid that you could infect your family?	No	47	7					43	11					45	9				
	Yes	728	214	0.538	0.216	1.341	0.183	673	269	0.674	0.311	1.463	0.319	682	260	0.479	0.211	1.087	0.078
Are you worried about the lack of PPE (Personal Protective Equipment)?	No	77	16					72	21					75	18				
	Yes	698	205	0.99	0.531	1.847	0.976	644	259	0.965	0.553	1.685	0.901	652	251	0.802	0.447	1.438	0.459
Do you feel less confident to treat critically ill patients?	No	424	90					393	121					400	114				
	Yes	351	131	0.657	0.48	0.901	**0.009**	323	159	0.72	0.542	0.958	**0.024**	327	155	0.638	0.476	0.856	**0.003**
In your opinion, in one day, what is the optimal time to work in the current situation?	less than 4 hours	58	21	0.698	0.329	1.482	0.350	56	23	1.204	0.600	2.417	0.601	58	21	1.265	0.618	2.587	0.520
	4 to 6 hours	200	67				0.777	183	84				0.652	192	75				0.970
	6 to 8 hours	395	98	0.867	0.457	1.645	0.662	368	125	1.118	0.603	2.074	0.724	364	129	1.240	0.658	2.334	0.506
	8 to 10 hours	102	22	0.749	0.401	1.399	0.365	88	36	0.897	0.491	1.638	0.723	92	32	1.188	0.641	2.202	0.584
	more than 10 hours	20	13	0.958	0.440	2.084	0.913	21	12	0.996	0.470	2.111	0.992	21	12	1.153	0.533	2.494	0.717
Do you have the opportunity of time to rest (physically or mentally) during the handling of this COVID-19 outbreak?	No	183	80					163	100					168	95				
	Yes	592	141	1.546	1.106	2.160	**0.011**	553	180	1.677	1.233	2.281	**0.001**	559	174	1.714	1.255	2.340	**0.001**
Are you satisfied with the hospital’s support for all of you during the handling of this COVID-19 outbreak?	No	78	52					77	53										
	Yes	697	169	2.483	1.649	3.739	**0.000**	639	227	1.831	1.235	2.715	**0.003**						
Do you need psychological help from the Counseling Unit/ Department of Psychiatry and Mental Health HTAR?	No	760	195					702	253					714	241				
	Yes	15	26	0.172	0.112	0.264	**0.000**	14	27	0.224	0.154	0.325	**0.000**	13	28	0.297	0.202	0.437	**0.000**

### Anxiety

For anxiety, 71.9% (716) were normal, 7.1% (71) were mild, 12.6% (125) were moderate, and 8.4% (84) were severe Therefore 71.9% (716) were normal and 28.1% (280) had anxiety (Table 2). This study showed that staff aged below 40 years old were twice more likely to have anxiety as compared to those aged 40 and above (AOR = 1.632; 95% CI: 1.141–2.334, p = 0.007) (Table 4). Staff with no involvement with COVID-19 patients were less likely to have anxiety (p = 0.118) as compared to those who had direct involvement with COVID-19 patients (AOR = 0.706; 95% CI: 0.503–0.990, p = 0.044). Staff who were confident in treating critical patients were less likely to have anxiety as compared to those who were less confident in treating critical patients (AOR = 0.720; 95% CI: 0.542–0.958, p = 0.024). Staff who have no time to rest during the COVID-19 pandemic are twice more likely to have anxiety as compared to those who have an adequate amount of rest (AOR = 1.677; 95% CI: 1.233–2.281, p = 0.001). Staff who were not satisfied with the hospital’s support were twice more likely to have anxiety as compared to those who are satisfied with the hospital during the COVID-19 pandemic (AOR = 1.831, 95% CI 1.235–2.715, p = 0.003). Staff who did not require psychological intervention provided by the hospital were less likely to have anxiety as compared to those who need of psychological support (AOR = 0.224; 95% CI: 0.154–0.325, p<0.05).

### Stress

For stress, majority, 73.0% (727) were normal, 18.7% (186) were mild, 6.1% (61) were moderate and 2.2% (22) were severe. Therefore, 73% (727) were normal and 27% (269) had stress. (Table 2). Among the staff, 320 staff (32.1%) had no contact with COVID-19 patients, 275 staff (27.6%) had direct contact with COVID-19 patients and 401 staff (40.3%) had indirect contact with COVID-19 patients (Table 3). Staff are worried to get infected with COVID-19 were twice more likely to have stress as compared to those who are not worried to get infected with COVID-19 (AOR = 1.963; 95% CI: 1.173–3.284, P = 0.010) (Table 4). Staff who were confident in treating critical patients were less likely to have anxiety as compared to those who were less confident in treating critical patients (AOR = 0.638; 95%CI: 0.476–0.856, p = 0.003) (Table 4).

## 4. Discussion

The present study examined the mental health toll of the COVID-19 pandemic on HCWs at the government hospital in Klang Valley, Malaysia. To our knowledge, this study is among the first to determine the prevalence and associated factors of depression, anxiety, and stress among HCWs in the government hospitals in Malaysia during the pandemic. The study was conducted during the first and second wave of the pandemic in Malaysia beginning of January 25 and February 27 2020 onwards [43]. The overall prevalence of depression, anxiety, and stress were 22.2%, 28.1%, and 27.0% respectively. These findings were consistent with another study reported by Woon LS et al., (2020) among university HCWs in Malaysia showed almost similar prevalence of depression (21.8%), anxiety (31.6%) and stress (29.1%) [44]. A review by Salari N et al., (2020) showed a high prevalence of psychological impacts with depression (24.3%; 95% CI 18.2–31.6%), anxiety (25.8%; 95% CI 20.5%-31.9%) and anxiety (45%; 95% CI 24.3–67.5%) among HCWs who treating COVID-19 patients [45]. A study by Woon et al., (2020) suggested that the psychological impact of the pandemic among HCWs persist even though the movement control order is lifted [44, 46].

### Depression

This study showed a majority of HCWs (89.6%) were concerned with their worries about the risk of being infected with COVID-19 while managing patients with COVID-19 and due to the lack of PPE supply to the HCWs (90.7%) as a result of this unprecedented pandemic. The finding from this study showed that HCWs were stressed by the fear of exposure to getting infected by COVID-19 patients due to the increasing trend of COVID-19 cases. A study by Joob B et al., (2020) reported a staff nurse contracted COVID-19 during managing a dengue patient in a Thai hospital although she addressed the precautionary measures during the pandemic COVID-19 [47]. Another study in Wuhan, China showed a large cluster of pneumonia patients and health professionals contracted COVID-19 in the same wards [48]. Being elderly is the greatest risk factor for mortality or morbidity of COVID-19 reported by Wu et al., 2020 who studied hospitalised patients in China [49]. Socio-demographic and underlying medical factors contribute to the risk of psychological disorders in the elderly during the pandemic [50]. Similar findings of higher psychological distress among older Jordanian HCWs during the COVID-19 pandemic were studied by Alnazly E et al., (2021) [51]. Working directly with Covid-19 patients was related overall to greater psychological distress than working indirectly. Ge et al., (2021) reported that transmission of COVID-19 among HCWs were common with index patient although they were clinically present without symptoms due to long-period exposure to close contacts [52]. This present study showed a positive association between depression and being a young HCWs (aged below 40 years old), namely fear of exposure to COVID-19 infection and being infected when managing COVID-19 patients during the pandemic. Furthermore, heavy workload and lack of coping with difficulties during the pandemic warrant social or psychological support from psychological services at the workplace.

### Anxiety

Our current study also showed that there is an association between inadequate rest from long hours of working shifts during the pandemic COVID-19 with psychological distress among HCWs. In a study by Subhas N et al., (2021), shift work consistently predicted anxiety among frontline healthcare workers [53]. These findings were replicated in another study, whereby shift work consistently predicted anxiety amongst frontline healthcare workers [54]. A study by Luceño-Moreno et al., (2020) suggested that HCWs who take a break from long working hours showed lower levels of stress [54]. de Sire A et al., (2021) did a survey among physical therapists related to work and healthcare issues related to COVID-19 showed the importance of a healthy psychosocial work environment to enhance job satisfaction of all health professionals to avoid burnout syndrome and avoid role conflict during the COVID-19 pandemic [55]. Besides breaks from long hours of working shifts, our study showed HCWs need psychological support during COVID-19 pandemic at working place. This can be done by support and empowerment through education and consultation for HCWs to overcome anxiety, stress and depression. Direct support from management can help the HCWs to develop a positive attitude and manage their work-related stress related [50, 56, 57]. HCWs need to feel safe and confident while working at health facilities or working areas where they will be protected at work [58].

### Stress

This present study showed a strong association between stress and feeling worried to get infected with COVID-19 among the staff. As a result, stress may suppress individual’s confidence in treating critical patients. In this sense, especially during pandemic situations, majority of our healthcare workers succumb with stress as an immediate effect on them. A previous study by Woon LS et al., (2020) reported fear of exposure to COVID-19 among healthcare workers at university hospitals in Malaysia [44]. Another study done by Alnazly et al., (2021) on Jordanian healthcare workers reported a positive association between fear and stress [51]. A review by Salari N et al., (2020) reported a high prevalence of stress among healthcare workers [21]. These highlighted the need of providing timely psychological support or treatment to healthcare workers who suffered psychological distress especially during pandemic to ensure the mental health state of healthcare workers is kept at optimal level.

## 5. Limitations

This study has some limitations. Firstly, the respondents recruited were all HCWs from a government hospital without any control group that was not involve at all with Covid-19 patients and this may not be able to generalise the result. Secondly, using psychometric questionnaires that were not designed to evaluate depression, anxiety and stress in the context of the pandemic do not reflect the true scenarios as psychological distress may have been undiagnosed. Thirdly, we didn’t conduct pre-test and post-test of DASS-21 after giving out the intervention. So, we couldn’t quantify the level of reduction of depression, anxiety and stress among the participants after prescribed the intervention.

Lastly, this current study reported the prevalence of depression, anxiety and stress should be interpreted with caution because all respondents were enrolled in a serial programme on Mental Health and Psychosocial Support in Covid-19 (MHPSS COVID-19). Thus, the findings could be lower than what would be reported among healthcare workers who are not enrolled in a similar programme. A longitudinal study involving all HCWs across all categories would have yielded better information since this study is only cross-sectional. Besides, details of personal and familial issues which could also affect the mental state of respondents were not collected. We didn’t consider ethnic group in further analysis since Malay-ethnic represent more than half of the population followed by the Chinese and Indians. We excluded non-malay speakers among HCWs since majority Malaysians is using common language which is the Malay Language benefited using the malay version of DASS-21. Those HCWs didn’t give their consent to undergo the survey, they can receive the psychological and emotional support in view of the importance of early mental health treatment. Nevertheless, this study provides unique opportunities for robust evaluation of mental health status among HCWs.

## 6. Conclusions

Our findings revealed the significant risk of adverse mental health outcomes for HCWs treating COVID-19 patients in hospital settings during the peak first and second wave of pandemic in Malaysia. Further research is important to understand the extent of the psychological impacts of pandemic on HCWs over time and identify effective psychological supports to meet their psychological needs and reduce the risk of long-term psychological damage in this COVID-19 pandemic and beyond.

## Supporting information

S1 Appendix Malay version of DASS-21. (TIFF)

## References

[1] Wang H, Li X, Li T, Zhang S, Wang L, Wu X, et al. The genetic sequence, origin, and diagnosis of SARS-CoV-2. European Journal of Clinical Microbiology & Infectious Diseases. 2020 Sep;39(9):1629–35. doi: 10.1007/s10096-020-03899-4 32333222 PMC7180649

[2] Guo YR, Cao QD, Hong ZS, Tan YY, Chen SD, Jin HJ, et al. The origin, transmission and clinical therapies on coronavirus disease 2019 (COVID-19) outbreak–an update on the status. Military Medical Research. 2020 Dec;7(1):1–0. doi: 10.1186/s40779-020-00240-0 32169119 PMC7068984

[3] Mohanty SK, Satapathy A, Naidu MM, Mukhopadhyay S, Sharma S, Barton LM, et al. Severe acute respiratory syndrome coronavirus-2 (SARS-CoV-2) and coronavirus disease 19 (COVID-19)–anatomic pathology perspective on current knowledge. Diagn Pathol 15, 103 (2020). doi: 10.1186/s13000-020-01017-8 32799894 PMC7427697

[4] Li Q, Guan X, Wu P, Wang X, Zhou L, Tong Y, et al. Early transmission dynamics in Wuhan, China, of novel coronavirus–infected pneumonia. New England journal of medicine. 2020 Jan 29. doi: 10.1056/NEJMoa2001316 31995857 PMC7121484

[5] Zhu N, Zhang D, Wang W, Li X, Yang B, Song J, et al. A novel coronavirus from patients with pneumonia in China, 2019. New England journal of medicine. 2020 Jan 24. doi: 10.1056/NEJMoa2001017 31978945 PMC7092803

[6] World Health Organization (WHO). Disease Outbreak News: Pneumonia of unknown cause–China. [internet]. Geneva: World Health Organization; 5 January 2020. [cited 2021 Sept 5]. Available from https://www.who.int/emergencies/disease-outbreak-news/item/2020-DON229.

[7] Asita E. COVID-19 Outbreak in Malaysia. Osong Public Health and Research Perspectives. 2020;11(3):93–100. doi: 10.24171/j.phrp.2020.11.3.08 32494567 PMC7258884

[8] Mohd Hanafiah K, Chang DW. Public knowledge, perception and communication behavior surrounding COVID-19 in Malaysia. ResearchGate. net. 2020. Available from https://www.researchgate.net/profile/Khayriyyah-Mohd-Hanafiah/publication/340568282_Public_knowledge_perception_and_communication_behavior_surrounding_COVID-19_in_Malaysia/links/5eaf9cc4299bf18b95948fb0/Public-knowledge-perception-and-communication-behavior-surrounding-COVID-19-in-Malaysia.pdf

[9] New Straits Times. PM’s Movement Control Order speech in English. [internet]. Malaysia. New Straits Times: Nation. 2020. 2020 March 17. [cited 2021 Sept 5]. Available from https://www.nst.com.my/news/nation/2020/03/575372/full-text-pms-movement-control-order-speech-english

[10] Azlan AA, Hamzah MR, Sern TJ, Ayub SH, Mohamad E. Public knowledge, attitudes and practices towards COVID-19: A cross-sectional study in Malaysia. Plos one. 2020 May 21;15(5): e0233668. doi: 10.1371/journal.pone.0233668 32437434 PMC7241824

[11] Mak IWC, Chu CM, Pan PC, Yiu MGC, Chan VL. Long-term psychiatric morbidities among SARS survivors. General hospital psychiatry. 2009 Jul 1;31(4):318–26. doi: 10.1016/j.genhosppsych.2009.03.001 19555791 PMC7112501

[12] Cheng SK-W, Tsang JS-K, Ku K-H, Wong C-W, Ng Y-K. Psychiatric complications in patients with severe acute respiratory syndrome (SARS) during the acute treatment phase: a series of 10 cases. The British Journal of Psychiatry. 2004 Apr;184(4):359–60. doi: 10.1192/bjp.184.4.359 15056583

[13] Cheng SK, Wong CW, Tsang J, Wong KC. Psychological distress and negative appraisals in survivors of severe acute respiratory syndrome (SARS). Psychological Medicine. 2004 Oct;34(7):1187–1195. doi: 10.1017/s0033291704002272 15697045

[14] Chua SE, Cheung V, Cheung C, McAlonan GM, Wong JWS, Cheung EPT, et al. Psychological effects of the SARS outbreak in Hong Kong on high-risk health care workers. The Canadian Journal of Psychiatry. 2004 Jun;49(6):391–3. doi: 10.1177/070674370404900609 15283534

[15] Chua SE, Cheung V, McAlonan GM, Cheung C, Wong JWS, Cheung EPT, et al. 2004. Stress and psychological impact on SARS patients during the outbreak. *The Canadian Journal of Psychiatry*, 49(6), pp.385–390. doi: 10.1177/070674370404900607 15283533

[16] Cheng SKW, Wong CW. Psychological intervention with sufferers from severe acute respiratory syndrome (SARS): lessons learnt from empirical findings. Clinical Psychology & Psychotherapy: An International Journal of Theory & Practice. 2005 Jan;12(1):80–6. doi: 10.1002/cpp.429

[17] Sim K, Chua HC. The psychological impact of SARS: a matter of heart and mind. Cmaj. 2004 Mar 2;170(5):811–2. doi: 10.1503/cmaj.1032003 14993176 PMC343855

[18] Medscape UK [internet]. UK: Medscape; 15 Apr 2020. New resources to help health care workers cope with COVID-19-related stress. [cited 2021 Sept 5]. Available from https://www.medscape.co.uk/viewarticle/new-resources-help-health-care-workers-cope-covid-19-related-2020a10013bk

[19] Wing YK, Ho MY. Mental health of patients infected with SARS. In Challenges of Severe Acute Respiratory Syndrome. Elsevier (Singapore) Pte Ltd; Hong Kong: 2006. p. 526–546. Available at https://hub.hku.hk/handle/10722/120196

[20] Moldofsky H, Patcai J. Chronic widespread musculoskeletal pain, fatigue, depression and disordered sleep in chronic post-SARS syndrome; a case-controlled study. BMC Neurol. 2011 Mar 24;11:37. doi: 10.1186/1471-2377-11-37 21435231 PMC3071317

[21] Salari N, Hosseinian-Far A, Jalali R, Vaisi-Raygani A, Rasoulpoor S, Mohammadi M, et al. Prevalence of stress, anxiety, depression among the general population during the COVID-19 pandemic: a systematic review and meta-analysis. Globalization and health. 2020 Dec;16(1):1–1. doi: 10.1186/s12992-020-00589-w 32631403 PMC7338126

[22] Wang C, Pan R, Wan X, Tan Y, Xu L, Ho CS, et al. Immediate psychological responses and associated factors during the initial stage of the 2019 coronavirus disease (COVID-19) epidemic among the general population in China. International journal of environmental research and public health. 2020 Jan;17(5):1729. doi: 10.3390/ijerph17051729 32155789 PMC7084952

[23] Qiu J, Shen B, Zhao M, Wang Z, Xie B, Xu Y. A nationwide survey of psychological distress among Chinese people in the COVID-19 epidemic: implications and policy recommendations. General psychiatry. 2020;33(2). doi: 10.1136/gpsych-2020-100213 32215365 PMC7061893

[24] McAlonan GM, Lee AM, Cheung V, Cheung C, Tsang KWT, Sham PC, et al. Immediate and sustained psychological impact of an emerging infectious disease outbreak on health care workers. The Canadian Journal of Psychiatry. 2007 Apr;52(4):241–7. doi: 10.1177/070674370705200406 17500305

[25] Rehman U, Shahnawaz MG, Khan NH, Kharshiing KD, Khursheed M, Gupta K, et al. Depression, anxiety and stress among Indians in times of Covid-19 lockdown. Community mental health journal. 2021 Jan;57(1):42–8. doi: 10.1007/s10597-020-00664-x 32577997 PMC7309680

[26] Elbay RY, Kurtulmuş A, Arpacıoğlu S, Karadere E. Depression, anxiety, stress levels of physicians and associated factors in Covid-19 pandemics. Psychiatry research. 2020 Aug 1;290:113130. doi: 10.1016/j.psychres.2020.113130 32497969 PMC7255248

[27] Pappa S, Ntella V, Giannakas T, Giannakoulis VG, Papoutsi E, Katsaounou P. Prevalence of depression, anxiety, and insomnia among healthcare workers during the COVID-19 pandemic: A systematic review and meta-analysis. Brain, behavior, and immunity. 2020 Aug 1;88:901–7. doi: 10.1016/j.bbi.2020.05.026 32437915 PMC7206431

[28] Vindegaard N, Benros ME. COVID-19 pandemic and mental health consequences: Systematic review of the current evidence. Brain, behavior, and immunity. 2020 Oct 1;89:531–42. doi: 10.1016/j.bbi.2020.05.048 32485289 PMC7260522

[29] Badahdah A, Khamis F, Al Mahyijari N, Al Balushi M, Al Hatmi H, Al Salmi I, et al. The mental health of health care workers in Oman during the COVID-19 pandemic. International Journal of Social Psychiatry. 2021 Feb;67(1):90–5. doi: 10.1177/0020764020939596 32635837 PMC8191150

[30] Lai J, Ma S, Wang Y, Cai Z, Hu J, Wei N, et al. Factors associated with mental health outcomes among health care workers exposed to coronavirus disease 2019. JAMA network open. 2020 Mar 2;3(3):e203976–. doi: 10.1001/jamanetworkopen.2020.3976 32202646 PMC7090843

[31] Zhang J, Lu H, Zeng H, Zhang S, Du Q, Jiang T, et al. The differential psychological distress of populations affected by the COVID-19 pandemic. Brain, behavior, and immunity. 2020 Jul;87:49. doi: 10.1016/j.bbi.2020.04.031 32304883 PMC7156946

[32] Taquet M, Luciano S, Geddes JR, Harrison PJ. Bidirectional associations between COVID-19 and psychiatric disorder: retrospective cohort studies of 62 354 COVID-19 cases in the USA. The Lancet Psychiatry. 2021 Feb 1;8(2):130–40. doi: 10.1016/S2215-0366(20)30462-4 33181098 PMC7820108

[33] Sanders JM, Monogue ML, Jodlowski TZ, Cutrell JB. Pharmacologic treatments for coronavirus disease 2019 (COVID-19): a review. Jama. 2020 May 12;323(18):1824–36. doi: 10.1001/jama.2020.6019 32282022

[34] Buheji M, Jahrami H, Dhahi AS. Minimising stress exposure during pandemics similar to COVID-19. International Journal of Psychology and Behavioral Sciences. 2020;10(1):9–16. doi: 10.5923/j.ijpbs.20201001.02

[35] Lazarus RS, Folkman S. Stress, appraisal and coping. Springer Publishing Company, Inc. 1984. ISBN 0-8261-4191-9. Available from https://www.academia.edu/37418588/_Richard_S_Lazarus_PhD_Susan_Folkman_PhD_Stress_BookFi_.

[36] American Psychiatric Association. Diagnostic and statistical manual of mental disorders: DSM-5. 5^th^ edition. American Psychiatric Publishing Inc. doi: 10.1176/a.bks.9780890425596

[37] Khan S, Siddique R, Li H, Ali A, Shereen MA, Bashir N, et al. Impact of coronavirus outbreak on psychological health. Journal of global health. 2020 Jun;10(1). doi: 10.7189/jogh.10.010331 32355556 PMC7180007

[38] Wittchen H-U. Generalized anxiety disorder: prevalence, burden, and cost to society. Depression and anxiety. 2002;16(4):162–71. doi: 10.1002/da.10065 12497648

[39] Roy-Byrne PP, Craske MG, Stein MB. Panic disorder. Lancet. 2006 Sep 16;368(9540):1023–32. doi: 10.1016/S0140-6736(06)69418-X 16980119

[40] Ramli M, Salmiah MA. Validation and psychometric properties of Bahasa Malaysia version of the Depression Anxiety and Stress Scales (DASS) among diabetic patients. Malaysian Journal of Psychiatry. 2009;18(2). Available from https://www.researchgate.net/publication/238675002_Validation_and_Psychometric_Properties_of_Bahasa_Malaysia_Version_of_the_Depression_Anxiety_And_Stress_Scales_DASS_Among_Diabetic_Patients

[41] Musa R, Fadzil MA, Zain Z. Translation, validation and psychometric properties of Bahasa Malaysia version of the Depression Anxiety and Stress Scales (DASS). ASEAN Journal of Psychiatry. 2007 Jan 1;8(2):82–9. Available from https://www.ramlimusa.com/wp-content/uploads/2.DASS_Musa_1_070917.pdf

[42] World Health Organization [Internet]. Geneva: World Health Organization; Mental health and psychosocial considerations during the COVID-19 outbreak; 18 March 2020 [cited 2021 Sept 5]; Available from https://apps.who.int/iris/bitstream/handle/10665/331490/WHO-2019-nCoV-MentalHealth-2020.1-eng.pdf?sequence=1&isAllowed=y

[43] Hashim JH, Adman MA, Hashim Z, Mohd Radi MF, Kwan SC. COVID-19 epidemic in Malaysia: epidemic progression, challenges, and response. Frontiers in public health. 2021 May 7;9:560592. doi: 10.3389/fpubh.2021.560592 34026696 PMC8138565

[44] Woon LS-C, Sidi H, Nik Jaafar NR, Abdullah MFIL. Mental health status of university healthcare workers during the COVID-19 pandemic: A post–movement lockdown assessment. International Journal of Environmental Research and Public Health. 2020 Jan;17(24):9155. doi: 10.3390/ijerph17249155 33302410 PMC7762588

[45] Salari N, Khazaie H, Hosseinian-Far A, Khaledi-Paveh B, Kazeminia M, Mohammadi M, et al. The prevalence of stress, anxiety and depression within front-line healthcare workers caring for COVID-19 patients: a systematic review and meta-regression. Human resources for health. 2020 Dec;18(1):1–4. doi: 10.1186/s12960-020-00544-1 33334335 PMC7745176

[46] Brooks SK, Webster RK, Smith LE, Woodland L, Wessely S, Greenberg N, et al. The psychological impact of quarantine and how to reduce it: rapid review of the evidence. The lancet. 2020 Mar 14;395(10227):912–20. doi: 10.1016/S0140-6736(20)30460-8 32112714 PMC7158942

[47] Joob B, Wiwanitkit V. COVID-19 in medical personnel: observation from Thailand. Journal of Hospital Infection. 2020 Apr 1;104(4):453. doi: 10.1016/j.jhin.2020.02.016 32114054 PMC7134413

[48] Wang D, Hu B, Hu C, Zhu F, Liu X, Zhang J, et al. Clinical characteristics of 138 hospitalized patients with 2019 novel coronavirus–infected pneumonia in Wuhan, China. Jama. 2020 Mar 17;323(11):1061–9. doi: 10.1001/jama.2020.1585 32031570 PMC7042881

[49] Wu C, Chen X, Cai Y, Xia J, Zhou X, Xu S, et al. Risk factors associated with acute respiratory distress syndrome and death in patients with coronavirus disease 2019 pneumonia in Wuhan, China. JAMA internal medicine. 2020 Jul 1;180(7):934–43. doi: 10.1001/jamainternmed.2020.0994 32167524 PMC7070509

[50] Webb LM, Chen CY. The COVID‐19 pandemic’s impact on older adults’ mental health: Contributing factors, coping strategies, and opportunities for improvement. International Journal of Geriatric Psychiatry. 2022 Jan;37(1). doi: 10.1002/gps.5647 34729802 PMC8646312

[51] Alnazly E, Khraisat OM, Al-Bashaireh AM, Bryant CL. Anxiety, depression, stress, fear and social support during COVID-19 pandemic among Jordanian healthcare workers. Plos one. 2021 Mar 12;16(3):e0247679. doi: 10.1371/journal.pone.0247679 33711026 PMC7954309

[52] Ge Y, Martinez L, Sun S, Chen Z, Zhang F, Li F, et al. COVID-19 transmission dynamics among close contacts of index patients with COVID-19: a population-based cohort study in Zhejiang province, China. JAMA Internal Medicine. 2021 Oct 1;181(10):1343–50. doi: 10.1001/jamainternmed.2021.4686 34424260 PMC8383161

[53] Subhas N, Pang NT-P, Chua W-C, Kamu A, Ho C-M, David IS, et al. The Cross-Sectional Relations of COVID-19 Fear and Stress to Psychological Distress among Frontline Healthcare Workers in Selangor, Malaysia. International Journal of Environmental Research and Public Health. 2021 Jan;18(19):10182. doi: 10.3390/ijerph181910182 34639482 PMC8508284

[54] Luceño-Moreno L, Talavera-Velasco B, García-Albuerne Y, Martín-García J. Symptoms of posttraumatic stress, anxiety, depression, levels of resilience and burnout in Spanish health personnel during the COVID-19 pandemic. International journal of environmental research and public health. 2020 Jan;17(15):5514. doi: 10.3390/ijerph17155514 32751624 PMC7432016

[55] de Sire A, Marotta N, Raimo S, Lippi L, Inzitari MT, Tasselli A, et al. Psychological Distress and Work Environment Perception by Physical Therapists from Southern Italy during COVID-19 Pandemic: The C.A.L.A.B.R.I.A Study. Int J Environ Res Public Health. 2021 Sep 14;18(18):9676. doi: 10.3390/ijerph18189676 34574600 PMC8465841

[56] Wong AH, Pacella-LaBarbara ML, Ray JM, Ranney ML, Chang BP. Healing the healer: protecting emergency health care workers’ mental health during COVID-19. Annals of emergency medicine. 2020 Oct 1;76(4):379–84. doi: 10.1016/j.annemergmed.2020.04.041 32534830 PMC7196406

[57] Chersich MF, Gray G, Fairlie L, Eichbaum QG, Mayhew S, Allwood B, et al. COVID-19 in Africa: care and protection for frontline healthcare workers. Globalization and health. 2020 Dec;16(1):1–6. doi: 10.1186/s12992-020-00574-3 32414379 PMC7227172

[58] Kursumovic E, Lennane S, Cook TM. Deaths in healthcare workers due to COVID‐19: the need for robust data and analysis. Anaesthesia. 2020 Aug 1. doi: 10.1111/anae.15116 32397005 PMC7272944

