# Supplementary material for: METER (Mental health emergency response) program: Findings of psychological impact status and factors associated with depression, anxiety and stress among healthcare workers in public hospital in Malaysia during the COVID-19 pandemic
Source: PLOS Glob Public Health. 2023 Apr 14;3(4):e0001823. doi: 10.1371/journal.pgph.0001823 (PMC10104317; doi:10.1371/journal.pgph.0001823)
Supplement: Attachment — Submitted filename: rebuttal letter.docx [file pgph.0001823.s002.docx]

Rebuttal letter

We thank the Academic Editor and four reviewers for their comments on our manuscript. Below is our response to each point raised by the academic editor and reviewers. We hope that we satisfyingly addressed them and that the manuscript will be suited for publication.

Sincerely,

On behalf of all authors

Dr Nor Asiah Muhamad

PGPH-D-22-00535
METER (Mental health Emergency Response) Program: Psychological Impact of the COVID-19 Pandemic on healthcare workers in a Tertiary Hospital in Klang Valley, Malaysia

| **No** | **Journal requirements** | **Author response** |
| --- | --- | --- |
| 1. | Please provide separate figure files in .tif or .eps format.  For more information about figure files please see our guidelines:  LINK  https://journals.plos.org/globalpublichealth/s/figures  https://journals.plos.org/globalpublichealth/s/figures#loc-file-requirements | All table 1, 2, 3, 4 was edited to comply with th format requirements. |
| **No** | **Editor Comments** | **Author response** |
|  | Please ensure native English speaker (an English editor) proofread the revised version of this paper to improve the readability and clarity of the message being communicated.  [Note: HTML markup is below. Please do not edit.] | Proofread the revised version done. |
| **No** | **Comments to the Author** |  |
| 1 | Does this manuscript meet PLOS Global Public Health’s [publication criteria](https://journals.plos.org/globalpublichealth/s/criteria-for-publication)? Is the manuscript technically sound, and do the data support the conclusions? The manuscript must describe methodologically and ethically rigorous research with conclusions that are appropriately drawn based on the data presented.  Reviewer #1: Yes  Reviewer #2: Yes  Reviewer #3: Yes  Reviewer #4: Yes |  |
| 2 | Has the statistical analysis been performed appropriately and rigorously?  Reviewer #1: Yes  Reviewer #2: Yes  Reviewer #3: Yes  Reviewer #4: Yes |  |
| 3 | Have the authors made all data underlying the findings in their manuscript fully available (please refer to the Data Availability Statement at the start of the manuscript PDF file)?  The [PLOS Data policy](https://journals.plos.org/globalpublichealth/s/data-availability) requires authors to make all data underlying the findings described in their manuscript fully available without restriction, with rare exception. The data should be provided as part of the manuscript or its supporting information, or deposited to a public repository. For example, in addition to summary statistics, the data points behind means, medians and variance measures should be available. If there are restrictions on publicly sharing data—e.g. participant privacy or use of data from a third party—those must be specified.  Reviewer #1: Yes  Reviewer #2: Yes  Reviewer #3: Yes  Reviewer #4: No |  |
| 4 | Is the manuscript presented in an intelligible fashion and written in standard English?  PLOS Global Public Health does not copyedit accepted manuscripts, so the language in submitted articles must be clear, correct, and unambiguous. Any typographical or grammatical errors should be corrected at revision, so please note any specific errors here.  Reviewer #1: Yes  Reviewer #2: No  Reviewer #3: Yes  Reviewer #4: No |  |
| 5 | Review Comments to the Author  Please use the space provided to explain your answers to the questions above. You may also include additional comments for the author, including concerns about dual publication, research ethics, or publication ethics. (Please upload your review as an attachment if it exceeds 20,000 characters)  Reviewer #1: Dear Authors,  The data presented was very extensive and could be simplified but my only fear is the meaning could be lost if the simplification was not done intelligibly. Having said that, the wealth of information does reflect on the targeted issue. However, the limitation is rather concerning. The design itself is notable sufficient without the mention of a bigger population that is not part of the actual study despite it could give a better reflection on the issue. But this is not the issue within the manuscript but rather to be pondered by the researcher. | We thank the reviewer #1 for comments on our manuscript. All tables have been simplified based on our appointed experts in the field. |
|  | Reviewer #2: Thanks for the opportunity to review this manuscript. This is an important topic that will allow policy makers and healthcare institutions to better support their staff, especially in outbreak situations. I have a few major comments below:  In general, the manuscript also requires further proofreading for grammar - I have not provided specific recommendations as there are errors throughout the manuscript. Given that PLOS Global Public Health does not copyedit accepted manuscripts, this would be a necessary major revision made by the authors. The team may consider getting a co-author to proofread the paper once again, or use software like Grammarly to assess the manuscript and create a revision. My comments below therefore reflect suggestions in terms of content, but not writing style.  ABSTRACT Lines 35-37: This statement should also be accompanied by the corresponding statistical output (adjusted odds ratio etc.)  INTRODUCTION Line 47: To be more specific, it was declared by the WHO as a public health emergency of international concern (PHEIC) Lines 60-62: This is vague - the authors could clarify what 'developing a lack of resilience' means because resilience is a psychological construct that should be described and further substantiated. Furthermore, are there specific 'various conditions' that the authors are referring to? Lines 62-64: This sentence also seems vague - is this referring to any data in particular? Lines 68-70: This doesn't seem relevant to the study given that the focus is on healthcare workers - unless the authors are making a link between HCWs as being at greatest risk for infection too and therefore being patients themselves? Lines 73-75: For these 'common disorders associated with the outbreak' - among whom would the authors be referring to? These survivors mentioned above? Lines 75-76: Same for here - among whom are these psychological sequelae present? Lines 76+: In general, this feels like a separate point - the data may refer more to the impact of the pandemic (movement control measures + everything else) rather than clinical co-morbidities. I feel like there is a conflation of these issues in the second paragraph and the authors should make an effort to separate them for clarity. Line 101: COVID-19 as a disease in patients? In other words, are the authors saying that getting infected with COVID-19 and dealing with it is a huge stressor in patients?  METHODS Line 146: What would a 'serial' programme be referring to? Please do clarify this as it seems slightly vague Lines 191-193: Would this mean that variables with p<0.05 at bivariable level were included in the multivariable analysis? The p-value at which statistical significance is taken at should be specified Otherwise, the methods are well-described  RESULTS Table 4: Given that the tables should be understood even when read without the main text, terms such as 'Involvement' could be expanded a little as it's hard to tell what this means (e.g. involvement in MPHSS? Or involvement in COVID care?) I know this is evident in the main text but does not work well as a standalone term in the table.  DISCUSSION Limitations: Given that the survey was done in Malay only, is this reflective of all HCWs in Malaysia? E.g. were the participants in MPHSS ethnically diverse? Limitations: The authors could also discuss briefly what this means for HCWs who are not linked to interventions - would it be that those who are surveyed have better outcomes already given that they were recruited in the context of an intervention? | Thank you to reviewer #2 for pointing these out for us to revise our manuscript.  ABSTRACT  Lines 35-37: the corresponding statistical output has been revised.  INTRODUCTION  Line 47: revision the highlighted done.  Lines 60-62: we removed this sentence.  Lines 62-64: we rephrased the sentence.  Lines 68-70: we rephrased the sentence.  Lines 73-75: we removed the sentence.  Lines 75-76: we rephrased the sentence.  Lines 76+: we rephrased the sentence.  Line 101:this finding from study in China among HCWs suffering psychological problems based on direct involvement with managing COVID-19 or indirectly.  Methods  Line 146: we rephrased the sentence.  Line 191-193: we specified the p value in the sentence.  Results  Table 4: details on ‘Involvement’ has been revised in the manuscript.  Discussion  Limitation in ethnicity we specified. The HCWs group who are not linked to intervention also we specified. |
|  | Reviewer #3: The manuscript was able to be understood well and it was written clearly to present the findings. However, I find that the repetitive nature of how the results were written make me confused. The discussion was well written and conclusion of the manuscript do depict the objective of the study. | We thank to reviewer #3 for comments on our manuscript. |
|  | Reviewer #4: Title of manuscript: METER (Mental health Emergency Response) Program: Psychological Impact of the COVID-19 Pandemic on healthcare workers in a Tertiary Hospital in Klang Valley, Malaysia  Thanks for asking me to review the manuscript titled, “METER (Mental health Emergency Response) Program: Psychological Impact of the COVID-19 Pandemic on healthcare workers in a Tertiary Hospital in Klang Valley,Malaysia”  The topic is very topical and I commend the authors for putting this together. Overall, the findings are important and of value to a national and international audience.  I think the paper would benefit from substantial editing by an English language editor to make it easier to understand and provide adequate feedback. There were a number of words that were missing and in some instances it was somewhat difficult understanding the information that was presented.  I also think that it might be beneficial to modify the title so that it aligns better with the results presented. Authors also need to re-arrange their results and discussion in line with the objectives. This will improve the flow and readability of the manuscript.  Specific issues are outlined below  Title Findings on the psychological status of the Healthcare workers prior to the pandemic were not presents making it difficult to make an assertion that their current psychological status was as a result of the pandemic.  I suggest a rephrasing of the title.  Abstract  Lines 21 – 23: “This study aims to identify the prevalence and factors of depression, anxiety and stress among HCWs in Malaysian healthcare facilities in the midst [of] the pandemic due to the SARS-CoV-2.”  Line 23: The word, “of”, is missing. Please see above and insert.  Lines 28 - 30: 28 Results: Of the1,300 staffs [who] attended the Mental Health and 29 Psychosocial Support in Covid-19 (MHPSS COVID-19) programme, 996 staffs (21.6% male, 30 78.4% female) completed the online survey (response rate: 76.6%). Result showed [that staff] aged] 31 above 40 years old were almost two times higher [please, replace the word “higher” with, “more likely”] to have anxiety (AOR=1.632; 95% CI=1.141- 32 2.334, p:0.007) and depression (AOR=1.637; 95% CI=1.1.06-2.423, p:0.014) as compared to 33 [staff who were] less than 40 years old.  Insert “who” in line 28. Lines 35 – 37: 35 “… … …HCWs 36 with stress, anxiety and depression showed less confident to treat critically ill patients and 37 need psychological help during outbreak”.  Please revise the statement (lines 35 – 37) above. Statement is not clear.  Conclusion: This study showed the importance of 38 a healthy psychosocial support to reduce psychological distress among HCWs when working 39 or coping during COVID-19 pandemic or outbreak.  Please, revise the statement above (Conclusion, lines 38 and 39). Statement is not clear.  Introduction  Lines 44 and 45: Use a consistent spelling for aetiology.  Line 47: due [to] its rapid spread and declared a pandemic on 12 March (4-6).  Insert missing word, “to” in the statement above.  Lines 59 – 60: Almost one-third of health care workers (HCWs) experiencing high levels of distress (17, 18).  Please change “experiencing”, to “experienced”   Lines 50 – 100: This paragraph is rather long and has more than one idea. I suggest that the paragraph be revised and split into two so each paragraph has one main idea.  Lines 101 to 102: COVID-19 is itself a huge stressor, as there are [change “are” to “were”] no existing medicine or vaccines during the start of the pandemic   Lines 121 to 122: 121 “Information on mental health status is 122 relevant for future programme on targeted group and evidence inform policy”.  Please revise the statement (lines 121 – 122) above. It is not clear.  Lines 134 to 142. Authors are describing the research objectives and hypothesis. The statements are not clear. I suggest the authors revise these objectives for clarity.  The Introduction needs substantial revision. I suggest that an English editor assist with revising the paragraphs, correcting mistakes and arranging the paragraphs such that each paragraph has one main idea.   The study objective and hypothesis stated in lines 130 to 137 are not exactly in line with the title which is, “Psychologic impact of COVID-19 on healthcare workers in a tertiary hospital in Klang Valley, Malaysia. Authors need to either revise the title or revise the study objectives and hypothesis. I suggest they review these after examining their results so they can decide on the title and objectives.  Lines 130 - 137 130 … … … Therefore, this study 132 aimed to determine the prevalence of depression, anxiety, and stress among HCWs in public 133 hospital in Malaysia. This study hypothesised a relevant prevalence rate for moderate to 134 severe psychological distress among HCWs. This study also aimed to examine the 135 relationships between sociodemographic traits, psychological characteristics and support 136 with depression, anxiety, and stress with the hypothesis that psychological characteristics and 137 support inversely associated with depression, anxiety, and stress.     Methods Requires revision. Authors may wish to review the Strobe guidelines and revise the methods section accordingly.  Some of the text uses red font, please correct.  Under data collection: The authors largely described the instruments here and not the data collection process.   Lines 165 to 169. This is a bit unclear. Authors mentioned that they used the modified version of Depression, Anxiety, and Stress Scale−21 (DASS– 21) – line 166. In line 169, they then mention using the BM DASS-21 scale. Is this the same instrument? Please use one consistent and correct term for the scale. It is also important to provide a brief explanation of the items in the scale and how participants are categorized as having depressive symptoms or not.  It isn’t clear what the psychological first aid tool was used for. I assumed it was to provide immediate help to study participants if they had complaints suggestive of psychological distress (as explained in lines 160 – 163). However, in lines 172 – 184, the authors mention that it was used as a screening tool.  Please see the description in the sub-section, “Psychological First Aid Screening Application and Reporting” lines 172 – 184.   Results  Lines 210 – 211  77.8% (775) were normal, 10.4% (104) were mild, 7.1% 210 (71) were moderate, and 4.6% (46) 211 were severe. Therefore, 77.8% (775) were normal and 22.2% (221) had depression.  I think the authors need to provide a brief description of the Malaysian version of the scale if this was what was used and also explain how the items in the scale are categorized. These could be attached as an appendix.  Lines 242 to 285 Findings on Multiple Logistic Regression between sociodemographic, psychological characteristics and support with Depression, 286 Anxiety and Stress 287 among healthcare workers are reported in this section.  Please re-order the report commencing with depression, anxiety and then stress (not stress, anxiety and depression, as it is currently reported). This improves the flow of the writing and reading, as the scale is called a DAS scale and presumably the questions are in this order.  As the participants were not asked about DAS symptoms prior to the pandemic and no results either from the participants’ records or other valid sources were explored and presented, it is not clear whether the prevalence reported was due to the pandemic, thus authors cannot conclude that the symptoms were related to the pandemic.  It might be better to title the current paper, “factors associated with depression, anxiety, and stress among HCWs in public hospital in Malaysia during the COVID-19 pandemic”.  This better reflects the results and is also in line with the study objective, typed in lines 131 to 133 131 Therefore, this study 132 aimed to determine the prevalence of depression, anxiety, and stress among HCWs in public 133 hospital in Malaysia.  This is also in line with the statement the authors made in the Discussion section: Lines 291 to 296. 291To our knowledge, this study is among the 292 first to determine the prevalence and associated factors of depression, anxiety, and stress 293 among HCWs in government hospital in Malaysia during the pandemic”  Lines 297 to 299 297These findings 298 suggest that the psychological impact of the pandemic among HCWs persist even though 299 movement control order has been eased at Malaysia.  The above statement is not clear. “these findings” …. Which findings are being referred to?  How do the findings, “suggest that the psychological impact of the pandemic among HCWs persist even though 299 movement control order has been eased at Malaysia”?  Lines 302 to 307.  302 The finding from this study showed that HCWs were stressed 303 from the fear of exposure to get infected from COVID-19 patients were significant. This finding 304 is similar to a study from other country that found HCWs have a higher risk of contracted with 305 COVID-19 at workplace. Study by Joob B et al (2020) reported a staff nurse contracted with 306 COVID-19 during managing dengue patient in a Thai hospital (45). Another study in Wuhan, 307 China showed a large cluster of pneumonia patients and health professionals   Please revise the statement above (lines 302 to 307) Please revise this statement as the meaning is not quite clear: “The finding from this study showed that HCWs were stressed from the fear of exposure to get infected from COVID-19 patients were significant.”  HCWs reporting a fear of getting exposed (line 303), is not the same as health workers actually getting infected. Hence authors’ statement that, “The finding from this study showed that HCWs were stressed from the fear of exposure to get infected from COVID-19 patients were significant. This finding is similar to a study from other country [which country is this?] that found HCWs have a higher risk of contracted with COVID-19 at workplace.  Authors could say that the health care workers were justified to be anxious about getting COVID-19 from the workplace as studies had reported that healthcare workers got the virus from the workplace.   Lines 309 to 311. The statements in these lines are not clear 309 HCWs above 40 years old in this study showed a significant psychological impact of anxiety 310 and depression. This could be a possible reason of being old workers is that the risk of 311 contracted with COVID-19 infection is higher than others  Lines 335 – 336 – Statement is not clear “Besides break from long hours of working shifts, our study showed HCWs need psychological support during COVID-19 pandemic at working place”  It isn’t clear how the study showed that, “showed HCWs need psychological support during COVID-19 pandemic at working place”. Did the authors ask questions about whether or not the participants wanted psychological support at the workplace during the pandemic? I don’t think I saw this result.    Limitations How would collecting data on effect of depression, anxiety and stress among the general population have improved the current study? How would conducting a longitudinal study involving all HCWs across all categories which yields better information? What would this information have been useful for?    Conclusion Lines 356 and 357 “This study showed the importance of a healthy psychosocial support to reduce psychological distress among HWCs when working or coping during COVID-19 pandemic or outbreak”.  I think authors should state that healthy psychosocial support could be useful in reducing psychological distress … … … This is because the study was not an intervention and authors did not have a comparison group of HCWs who did not receive psychological support. | Thank you to reviewer #4 for pointing this out for us to revise our manuscript.  Proofread the revised version done.  Title:  We rephrased the title.  Abstract  Lines 21-23: we added [of] as suggested.  Lines 28-30: We added [who], [that staff], [more likely], [staff who were] as suggested.  Lines 35-37: We amended the sentence by putting AOR as suggested reviewer #2.for each psychological illnesses.  Conclusion: We rephrased the sentence.  Introduction  Lines 44-45: We revised it using the consistent spelling for aetiology.  Line 47: We added [to] to the sentence.  Line 59-60: We amended the word [experiencing] to [experienced].  Line 50-100: We revised and split into three paragraphs  Line 101-102: we changed word [are] to [were].  Line 121-122: We removed the sentence.  Line 134-142: We removed the sentence.  We amended the related paragraph.  We revised the title.  We rephrased the sentence.  Methods  We rephrased the sentence.  We changed the colour.  We rephrased the sentence.  Line 165-169: We corrected the version.  We put up more info on this under the ‘Psychological First Aid Screening Application and Reporting’.  We put up in the earlier part.  Results  We put up the malay version of DASS-21 in the appendix.  We put up the re-order with depression, anxiety and stress.  We rephrased the title.  Line 297-299: We rephrased the sentence.  Line 302-307: We rephrased the sentence.  Line 309 to 311: we rephrased the sentence.  Line 335-336: we rephrased the sentence.  In table 4: there was a question asking about ‘Do you need psychological help from the Counseling Unit / Department of Psychiatry and Mental Health HTAR?’  Limitations: we removed the sentence.  we rephrased the sentence.  Conclusion:  Line 356-357: we rephrased the sentence. |
