# Supplementary material for: METER (Mental health emergency response) program: Findings of psychological impact status and factors associated with depression, anxiety and stress among healthcare workers in public hospital in Malaysia during the COVID-19 pandemic
Source: PLOS Glob Public Health. 2023 Apr 14;3(4):e0001823. doi: 10.1371/journal.pgph.0001823 (PMC10104317; doi:10.1371/journal.pgph.0001823)
Supplement: Attachment — Submitted filename: rebuttal letter PGPH-D-22-00535 R1_Dec 2022.docx [file pgph.0001823.s003.docx]

Rebuttal letter

Manuscript Number: PGPH-D-22-00535R1

Full Title: METER (Mental Health Emergency Response) Program: Findings of Psychological Impact Status and Factors Associated with Depression, Anxiety and Stress Among Healthcare Workers in Public Hospital in Malaysia during the COVID-19 pandemic

Joel Msafiri Francis, MD, MS, PhD

Academic Editor

PLOS Global Public Health

We sincerely appreciate all your valuable comments and suggestions from the Academic Editor and four reviewers which helped us in improving the quality of the manuscript. Below is our response to each point raised by the academic editor and reviewers. We hope that we satisfyingly addressed them and that the manuscript will be suited for publication.

Sincerely,

On behalf of all authors

Dr Nor Asiah Muhamad

PGPH-D-22-00535R1
Full Title: METER (Mental health Emergency Response) Program: Psychological Impact of the COVID-19 Pandemic on healthcare workers in a Tertiary Hospital in Klang Valley, Malaysia

| **No** | **Journal requirements** | **Author response** |
| --- | --- | --- |
| 1. | Please review your reference list to ensure that it is complete and correct. If you have cited papers that have been retracted, please include the rationale for doing so in the manuscript text, or remove these references and replace them with relevant current references. Any changes to the reference list should be mentioned in the rebuttal letter that accompanies your revised manuscript. If you need to cite a retracted article, indicate the article’s retracted status in the References list and also include a citation and full reference for the retraction notice. | All references have been reviewed. |
| **No** | **Editor Comments** | **Author response** |
| 1. | Additional Editor Comments (if provided):  Thanks for addressing the previous comments and suggestions. In addition to the reviewers comments – I strongly suggest that you proofread the manuscript to improve the flow of ideas and readability. |  |
| **No** | **Comments to the Author** |  |
| 1 | 1. If the authors have adequately addressed your comments raised in a previous round of review and you feel that this manuscript is now acceptable for publication, you may indicate that here to bypass the “Comments to the Author” section, enter your conflict of interest statement in the “Confidential to Editor” section, and submit your “Accept” recommendation.   Reviewer #1: All comments have been addressed  Reviewer #2: All comments have been addressed  Reviewer #3: All comments have been addressed  Reviewer #4: (No Response) |  |
| 2 | Does this manuscript meet PLOS Global Public Health’s [publication criteria](https://journals.plos.org/globalpublichealth/s/criteria-for-publication)? Is the manuscript technically sound, and do the data support the conclusions? The manuscript must describe methodologically and ethically rigorous research with conclusions that are appropriately drawn based on the data presented.  Reviewer #1: Yes  Reviewer #2: Yes  Reviewer #3: Yes  Reviewer #4: Yes |  |
| 3 | Has the statistical analysis been performed appropriately and rigorously?  Reviewer #1: Yes  Reviewer #2: Yes  Reviewer #3: Yes  Reviewer #4: Yes |  |
| 4 | Have the authors made all data underlying the findings in their manuscript fully available (please refer to the Data Availability Statement at the start of the manuscript PDF file)?  The [PLOS Data policy](https://journals.plos.org/globalpublichealth/s/data-availability) requires authors to make all data underlying the findings described in their manuscript fully available without restriction, with rare exception. The data should be provided as part of the manuscript or its supporting information, or deposited to a public repository. For example, in addition to summary statistics, the data points behind means, medians and variance measures should be available. If there are restrictions on publicly sharing data—e.g. participant privacy or use of data from a third party—those must be specified.  Reviewer #1: Yes  Reviewer #2: Yes  Reviewer #3: Yes  Reviewer #4: No |  |
| 5 | Is the manuscript presented in an intelligible fashion and written in standard English?  PLOS Global Public Health does not copyedit accepted manuscripts, so the language in submitted articles must be clear, correct, and unambiguous. Any typographical or grammatical errors should be corrected at revision, so please note any specific errors here.  Reviewer #1: Yes  Reviewer #2: No  Reviewer #3: Yes  Reviewer #4: (No Response) |  |
| **6.** | **Reviewer comments to Author** | **Author response** |
|  | Reviewer #1:  I do not have further comments but the revision done by the author to fulfil the requirements of the second reviewer I deem as sufficiently acceptable. It has also answered my previous concerns better. | Thank to Reviewer #1 for your time and effort in reviewing our manuscript. |
|  | Reviewer #2:  Thank you for addressing the comments. I believe the manuscript has greater clarity as a result. I'd suggest one more proofread for language as several sentences are not phrased correctly or are unclear. For example, in lines 84-86:  "Healthcare workers not only engage directly or indirectly with the treatment of infected patients but also the risk of being infected themselves, fear of transmission to family members, being stigmatized, rejected and working under stressful pressures."  In this sentence, while grammatically somewhat correct, the style makes it difficult to read and there is lack of clarity. A better phrasing would be:  "Healthcare workers not only engage with the treatment of infected patients, but are also a risk of being infected themselves. Apart from the risk of infection, they face additional stressors, such as the fear of transmitting COVID-19 to family members, being stigmatized or rejected by others based on their occupation, and working under highly stressful conditions."  I would suggest doing another proofread accordingly. | Thank to Reviewer #2 for pointing this out for us to revise our manuscript.  We have done the proofread too.  We have revised the sentences in lines 84-86 as suggested by Reviewer #2 (refer lines 85-89). |
|  | Reviewer #3:  It is a good study that is rarely done. However, it will good to capture more data from the professional groups like doctors. | Thank to Reviewer #3 for your time and effort in reviewing our manuscript. |
|  | Reviewer #4:  The authors have considerably improved the presentation of their findings, however, the manuscript still requires considerable editing especially as the journal will not be editing the manuscript before publishing. It was still rather difficult fully understanding some sections, especially the discussion, conclusion and limitation.  A few examples of statements that require correction are below:  Abstract HCWs with stress (AOR=0.638; 95% CI of 0.476 - 0.856, p= 0.003), anxiety (AOR=0.720; 95% CI 0.542 - 0.958, p= 0.024) and depression (AOR=0.657; 95% CI 0.480- 0.901, p= 0.009) showed less confident to treat critically ill patients and need psychological help during outbreak.  Please change, “confident”, to “confidence”  Introduction - The statement below is not clear. Was COVID-19 detected by or among three Chinese nationals? Please cross-check and correct as required. “In Malaysia, the first case of COVID-19 was detected by three Chinese nationals on 25 January 2020 …”  - Please write out the full meaning of SARS as it appears it is occurring for the first time here. Line 61: “… … outbreak of the 21st century, SARS”  - Please replace, “the hospital” with some concise details about the hospital e.g., was this a primary care hospital? In which city or country? “A study conducted in the hospital during the SARS”  - Please rephrase the sentence below: “Another finding Mak et al.’s paper found that the cumulative incidences of mental health disorders were 58.9% after the SARS outbreak”  - Lines 97 to 101. Please rephrase the sentence below “Further importance in early detection and treatment of psychiatric disorders during the Covid-19 outbreak was shown in a large, retrospective cohort study involving 62,354 participants in the United States which reported bidirectional associations in terms of the development of psychiatric disorders and acquiring Covid-19 infection (32).  - Authors are encouraged to discuss depression, anxiety and stress in the same order throughout the manuscript for consistence. The title is depression, anxiety and stress, hence it improves readability of this ordering is maintained, as much as possible.  - The introduction is an improvement on the previous version that was submitted, but it still requires editing.  Materials and methods This is an improvement on the previous version. The current version however still requires editing. Typically, this section begins with a description of the study area and not the ethical issues. Authors should please review the Strobe guidelines for writing observational studies and other manuscripts published in the journal.  - Lines 172 to 176 are repeated in Lines 184 to 186 “A Psychological First Aid (PFA) form was distributed online through a google form as a first aid screening which was sent out via QR code and electronic messaging system to all participants in the programme.”  - Lines 204 to 206 Please explain why these variables were included in the adjusted model. “Age, gender, and job category were predetermined to enter into the adjusted model, regardless of their bivariate association with each outcome”  - Data collection The sequence is a bit confusing. Were all the study participants provided with a mental health intervention before the study? If yes this needs to be known.  I think that the fact that all participants received a mental health intervention has introduced a bias into the study. The intervention would result in a lower level of depression, anxiety and stress. Authors need to discuss this in their limitations section.  Results - Please change staffs to staff in the opening sentence and in other sentences where “staffs” is used instead of “staff”.  - Delete the word, “staff”, highlighted below. It is not necessary to have it here “Of these, 996 staffs completed the online survey which gave a response rate of 76.6%”.  - The tables might need to be formatted in line with the journal guidelines. Please review other published papers for this.  - Line 245: Please change “staffs” to “staff”. Also change this in other sections in the manuscript.  - Lines 245 to 287. Please summarize the information in table 4. Too much detail is currently presented.  - Lines 299 and 300 Please delete, “for” in the sentence below “A review by Salari N et al (2020) showed a high prevalence of psychological impacts among HCWs who treat for COVID-19 patients (44).”  - Lines 306 to 308  Please revise the statement below. It is currently difficult to understand and not clear what point it is supporting or refuting.  “A study by Joob B et al (2020) reported a staff nurse contracted with COVID-19 during managing a dengue patient in a Thai hospital”  - Lines 311 to 313 Please revise the statement below. The meaning is unclear.  “HCWs above 40 years old in this study showed a high psychological impact of anxiety and depression. This could be a possible reason of being old workers is that the risk of contracting with COVID-19 infection is higher than others (47)”.  - Lines 316 to 319 Statement below is unclear. Please revise  “This is possibly due to the higher risk of contracted COVID-19 infection among index patients with COVID-19. Ge Y et al (2021) reported a greater potential of contracting with COVID-19 patients with quantity of exposure to a patient with COVID-19 among those close contacts (49).”  - Lines 320 to 320 The current study is a cross-sectional study and not able to demonstrate “cause and effect”, hence the claim below can not be made. Authors should please rephrase to indicate the association that they might have observed.  “Our study also showed that HCWs have psychological distress due to inadequate rest from long hours of working shifts during pandemic COVID-19”.  - Lines 323 to 326 is repeated in lines 328 to 331  “… emphasise the importance of maintaining standards in medical centres to foster shorter shifts and ensure enough resting periods to reduce the risk and vulnerability of HCWs against psychological burden and professional burnout in critical care scenarios”.  The discussion, conclusions and limitations require editing to improve understanding by the reader.  Conclusion  - Lines 362 to 366” The first few sentences (see below) are not clear  “HCWs face considerable challenges during the pandemic. This study showed the importance of psychosocial and emotional support to reduce psychological distress among HWCs when working or coping during COVID-19 pandemic or outbreak. This is because the study was not an intervention and authors did not have a comparison group of HCWs who did not receive psychological support”.  Authors need to read the manuscript carefully and address all grammatical issues to improve the manuscript. | Thank to Reviewer #4 for pointing this out for us to revise our manuscript.  We have changed the confident to confidence as suggested by Reviewer #4 (refer line 39).  The statement has been revised (refer lines 53-55).  We wrote out the full meaning of “SARS” (refer line 62).  We revised “the hospital” with more details about the hospital. (refer line 65).  We rephrased the sentence (refer lines 76 to 77).  We rephrased the sentence in Lines 97 to 101 (refer lines 100 to 103).  We reorganised “depression”, “anxiety” and “stress” in the same order in the chapter [Result] and [Discussion].  We revised the introduction.  Materials and methods:  We revised the sentence using Strobe guidelines for writing observational studies and other manuscripts published in the journal.  We rephrased the sentence in lines 172 to 176 and in line 186.  Lines 204 to 206: we removed this sentence.  Data collection: We revised the sentence.  Explanation: All the participants who agreed to participate in this study were provided with a mental health intervention after screening. Those HCWs didn’t agree to participate in this study, were provided with mental health intervention in view of the importance of early mental health treatment. We didn’t conduct pre-test and post-test of DASS-21 after giving out the intervention. So, we couldn’t quantify the level of reduction of depression, anxiety and stress among the participants. We add on this issue in the limitation.  Results: we changed “staffs” to “staff” in all sentences.  We removed “staff” from this sentence.  We revised the table 4 according to journal guidelines (refer line 282).  Line 245: We changed “staffs” to “staff” in this sentence as well in other sections in the manuscript.  Line 245 to 287: We summarized the information in table 4 (refer line 282).  Line 299 and 300: We deleted word “for” from the sentence (refer line 296).  Line 306 to 308: We revised the sentence (refer line 304 to 307).  Line 311 to 313: We revised the statement (refer line 309 to 310).  Line 316 to 319: We revised the statement (refer line 315 to 317).  Line 320: We rephrased the statement (refer line 324 to 326).  We removed both sentences.  We revised the discussion, conclusions and limitations.  Line 362 to 366: We revised the sentence to make it clearer (refer line 374 to 379).  We proofread to address all grammatical issues in this manuscript. |
| 7. | PLOS authors have the option to publish the peer review history of their article ([what does this mean?](https://journals.plos.org/globalpublichealth/s/editorial-and-peer-review-process#loc-peer-review-history)). If published, this will include your full peer review and any attached files.  **Do you want your identity to be public for this peer review?** If you choose “no”, your identity will remain anonymous but your review may still be made public.  For information about this choice, including consent withdrawal, please see our [Privacy Policy](https://www.plos.org/privacy-policy).  Reviewer #1: **Yes:** Dr Mohammad Zabri Johari  Reviewer #2: **Yes:** TAN KAY JIN RAYNER  Reviewer #3: **Yes:** Mohd Fadzli Mohamad Isa  Reviewer #4: No |  |
