# Supplementary material for: METER (Mental health emergency response) program: Findings of psychological impact status and factors associated with depression, anxiety and stress among healthcare workers in public hospital in Malaysia during the COVID-19 pandemic
Source: PLOS Glob Public Health. 2023 Apr 14;3(4):e0001823. doi: 10.1371/journal.pgph.0001823 (PMC10104317; doi:10.1371/journal.pgph.0001823)
Supplement: Attachment — Submitted filename: PGPH-D-22-00535R2_PLOS 022023.docx [file pgph.0001823.s004.docx]

| Date: | Feb 08 2023 12:02PM |
| --- | --- |
| To: | "NOR ASIAH MUHAMAD" norasiahdr@gmail.com;drnorasiah@moh.gov.my |
| cc: | joelmfrancis@gmail.com |
| From: | "PLOS Global Public Health" globalpubhealth@plos.org |
| Subject: | PLOS Global Public Health Decision: Revision required [PGPH-D-22-00535R2] |

PGPH-D-22-00535R2
METER (Mental health Emergency Response) Program: Findings of Psychological Impact Status and factors associated with depression, anxiety and stress among healthcare workers in public hospital in Malaysia during the COVID-19 pandemic
PLOS Global Public Health

Dear Dr. MUHAMAD,

Thank you for submitting your manuscript to PLOS Global Public Health. After careful consideration, we feel that it has merit but does not fully meet PLOS Global Public Health’s publication criteria as it currently stands. Therefore, we invite you to submit a revised version of the manuscript that addresses the points raised during the review process.

Please submit your revised manuscript by **Mar 10 2023 11:59PM**. If you will need more time than this to complete your revisions, please reply to this message or contact the journal office at globalpubhealth@plos.org. When you're ready to submit your revision, log on to https://www.editorialmanager.com/pgph/ and select the 'Submissions Needing Revision' folder to locate your manuscript file.

Please include the following items when submitting your revised manuscript:

- A rebuttal letter that responds to each point raised by the editor and reviewer(s). You should upload this letter as a separate file labeled 'Response to Reviewers'.
- A marked-up copy of your manuscript that highlights changes made to the original version. You should upload this as a separate file labeled 'Revised Manuscript with Track Changes'.
- An unmarked version of your revised paper without tracked changes. You should upload this as a separate file labeled 'Manuscript'.

Guidelines for resubmitting your figure files are available below the reviewer comments at the end of this letter.

We look forward to receiving your revised manuscript.

Kind regards,

Joel Msafiri Francis, MD, MS, PhD
Academic Editor
PLOS Global Public Health

Journal Requirements:

Please review your reference list to ensure that it is complete and correct. If you have cited papers that have been retracted, please include the rationale for doing so in the manuscript text, or remove these references and replace them with relevant current references. Any changes to the reference list should be mentioned in the rebuttal letter that accompanies your revised manuscript. If you need to cite a retracted article, indicate the article’s retracted status in the References list and also include a citation and full reference for the retraction notice.

Additional Editor Comments (if provided):

It would be helpful to address the final minor issues raised by the reviewers.


[Note: HTML markup is below. Please do not edit.]

Reviewers' comments:

Reviewer's Responses to Questions

Comments to the Author

1. If the authors have adequately addressed your comments raised in a previous round of review and you feel that this manuscript is now acceptable for publication, you may indicate that here to bypass the “Comments to the Author” section, enter your conflict of interest statement in the “Confidential to Editor” section, and submit your "Accept" recommendation.

Reviewer #2: All comments have been addressed

Reviewer #4: (No Response)

2. Does this manuscript meet PLOS Global Public Health’s [publication criteria](https://journals.plos.org/globalpublichealth/s/criteria-for-publication)? Is the manuscript technically sound, and do the data support the conclusions? The manuscript must describe methodologically and ethically rigorous research with conclusions that are appropriately drawn based on the data presented.

Reviewer #2: Yes

Reviewer #4: Yes

3. Has the statistical analysis been performed appropriately and rigorously?

Reviewer #2: Yes

Reviewer #4: Yes

4. Have the authors made all data underlying the findings in their manuscript fully available (please refer to the Data Availability Statement at the start of the manuscript PDF file)?

The [PLOS Data policy](https://journals.plos.org/globalpublichealth/s/data-availability) requires authors to make all data underlying the findings described in their manuscript fully available without restriction, with rare exception. The data should be provided as part of the manuscript or its supporting information, or deposited to a public repository. For example, in addition to summary statistics, the data points behind means, medians and variance measures should be available. If there are restrictions on publicly sharing data—e.g. participant privacy or use of data from a third party—those must be specified.

Reviewer #2: Yes

Reviewer #4: Yes

5. Is the manuscript presented in an intelligible fashion and written in standard English?

PLOS Global Public Health does not copyedit accepted manuscripts, so the language in submitted articles must be clear, correct, and unambiguous. Any typographical or grammatical errors should be corrected at revision, so please note any specific errors here.

Reviewer #2: No

Reviewer #4: Yes

6. Review Comments to the Author

Please use the space provided to explain your answers to the questions above. You may also include additional comments for the author, including concerns about dual publication, research ethics, or publication ethics. (Please upload your review as an attachment if it exceeds 20,000 characters)

Reviewer #2: Thanks for the revisions.

While the authors have mentioned that a proofread had been completed in their response, I am unfortunately still finding grammatical errors in the writing. For example:

"The first confirmed case of COVID-19 in Malaysia was detected among three Chinese nationality on 25 January 2020 who previously had close contact with person infected with COVID-19 in Singapore."

The phrase "three Chinese nationality" has no noun in it, and therefore is an incorrect sentence.

I believe that the authors need to engage an external proofreader to correct these errors, as these errors have persisted in the past iterations/revisions.

Reviewer #4: This is a greatly improved version. There are very few minor edits for which I have provided suggestions in the uploaded copy of the manuscript. The statements requiring editing are highlighted in blue to make it easy for authors to implement the very minor corrections.

Once these corrections are made, I recommend accepting the publication

7. PLOS authors have the option to publish the peer review history of their article ([what does this mean?](https://journals.plos.org/globalpublichealth/s/editorial-and-peer-review-process#loc-peer-review-history)). If published, this will include your full peer review and any attached files.


Do you want your identity to be public for this peer review? If you choose “no”, your identity will remain anonymous but your review may still be made public.


For information about this choice, including consent withdrawal, please see our [Privacy Policy](https://www.plos.org/privacy-policy).

Reviewer #2: Yes: Tan Kay Jin Rayner

Reviewer #4: No

[NOTE: If reviewer comments were submitted as an attachment file, they will be attached to this email and accessible via the submission site. Please log into your account, locate the manuscript record, and check for the action link "View Attachments". If this link does not appear, there are no attachment files.]

While revising your submission, please upload your figure files to the Preflight Analysis and Conversion Engine (PACE) digital diagnostic tool, https://pacev2.apexcovantage.com/. PACE helps ensure that figures meet PLOS requirements. To use PACE, you must first register as a user. Registration is free. Then, login and navigate to the UPLOAD tab, where you will find detailed instructions on how to use the tool. If you encounter any issues or have any questions when using PACE, please email PLOS at figures@plos.org. Please note that Supporting Information files do not need this step.

PGPH-D-22-00535R2

METER (Mental Health Emergency Response) Program: Findings of Psychological Impact Status and Factors Associated with Depression, Anxiety and Stress Among Healthcare Workers in Public Hospital in Malaysia during the COVID-19 pandemic

| No | Journal requirements | Author response |
| --- | --- | --- |
|  | Please review your reference list to ensure that it is complete and correct. If you have cited papers that have been retracted, please include the rationale for doing so in the manuscript text, or remove these references and replace them with relevant current references. Any changes to the reference list should be mentioned in the rebuttal letter that accompanies your revised manuscript. If you need to cite a retracted article, indicate the article’s retracted status in the References list and also include a citation and full reference for the retraction notice. | We reviewed all reference list.  We updated the reference no. 3, 9, 18, 19 |
| No | Editor Comments | Author response |
|  | It would be helpful to address the final minor issues raised by the reviewers. | We addressed the final minor issues raised by the reviewers. |
| No | Reviewers' Comments | Author response |
| 1. | If the authors have adequately addressed your comments raised in a previous round of review and you feel that this manuscript is now acceptable for publication, you may indicate that here to bypass the “Comments to the Author” section, enter your conflict of interest statement in the “Confidential to Editor” section, and submit your "Accept" recommendation.  Reviewer #2: All comments have been addressed  Reviewer #4: (No Response) | Thank to Reviewer #2 for your time and effort in reviewing our manuscript. |
| 2. | Does this manuscript meet PLOS Global Public Health’s [publication criteria](https://journals.plos.org/globalpublichealth/s/criteria-for-publication)? Is the manuscript technically sound, and do the data support the conclusions? The manuscript must describe methodologically and ethically rigorous research with conclusions that are appropriately drawn based on the data presented.  Reviewer #2: Yes  Reviewer #4: Yes | Thank to Reviewer #2 & #4 for your time and effort in reviewing our manuscript. |
| 3. | Has the statistical analysis been performed appropriately and rigorously?  Reviewer #2: Yes  Reviewer #4: Yes | Thank to Reviewer #2 & #4 for your time and effort in reviewing our manuscript. |
| 4. | Is the manuscript presented in an intelligible fashion and written in standard English?  PLOS Global Public Health does not copyedit accepted manuscripts, so the language in submitted articles must be clear, correct, and unambiguous. Any typographical or grammatical errors should be corrected at revision, so please note any specific errors here.  Reviewer #2: No  Reviewer #4: Yes | We sent the manuscript for proofread. |
| 5. | Have the authors made all data underlying the findings in their manuscript fully available (please refer to the Data Availability Statement at the start of the manuscript PDF file)?  The [PLOS Data policy](https://journals.plos.org/globalpublichealth/s/data-availability) requires authors to make all data underlying the findings described in their manuscript fully available without restriction, with rare exception. The data should be provided as part of the manuscript or its supporting information, or deposited to a public repository. For example, in addition to summary statistics, the data points behind means, medians and variance measures should be available. If there are restrictions on publicly sharing data—e.g. participant privacy or use of data from a third party—those must be specified.  Reviewer #2: Yes  Reviewer #4: Yes |  |
| 6. | Review Comments to the Author | Author response |
|  | Reviewer #2: Thanks for the revisions.  While the authors have mentioned that a proofread had been completed in their response, I am unfortunately still finding grammatical errors in the writing. For example:  "The first confirmed case of COVID-19 in Malaysia was detected among three Chinese nationality on 25 January 2020 who previously had close contact with person infected with COVID-19 in Singapore."  The phrase "three Chinese nationality" has no noun in it, and therefore is an incorrect sentence.  I believe that the authors need to engage an external proofreader to correct these errors, as these errors have persisted in the past iterations/revisions. | We revised the sentence:  [The first confirmed case of COVID-19 in Malaysia was detected among three Chinese nationality on 25 January 2020 who previously had close contact with people infected with COVID-19 in Singapore] to  [The first confirmed case of COVID-19 in Malaysia was detected among three Chinese nationals on 25 January 2020 who previously had close contact with people infected with COVID-19 in Singapore] |
|  | Reviewer #4: This is a greatly improved version. There are very few minor edits for which I have provided suggestions in the uploaded copy of the manuscript. The statements requiring editing are highlighted in blue to make it easy for authors to implement the very minor corrections.  Once these corrections are made, I recommend accepting the publication  Line 54: “Chinese nationality”  Line 55: “contact with person”  Line 65: “in the large teaching hospital”  Line 78: “the common psychological”  Line 103-104: “meaning not clear”  Line 159-160: need to state in the study limitations that the prevalence of depression, anxiety and stress obtained in the current study should be interpreted with caution because all respondents were enrolled in a serial programme on Mental Health and Psychosocial Support in Covid-19 (MHPSS COVID-19). Thus the findings could be lower than what would be reported among healthcare workers who are not enrolled in a similar programme.  Line 178: “The malay”  Line 282: “The present study examines”  Line 285: “in the government hospital”  Line 293: Typically authors’ initials are not mentioned when citing publications within the main body of the manuscript.  Line 298: “the risk to be infected”  Line 315: “depression with being young HCWs”  Line 338-3340: Please revise these two sentences “This present study showed a strong association between the feeling worry to get infected with COVID-19 stress and stress. Another notes, there is an association between feeling less confident individual's confidence in treating critical patients and stress too.”  Line 374: Please change “documenting” to “revealed”. | We revised according to Reviewer #4 suggestions:  Line 54: change “Chinese nationality” to “Chinese nationals”.  Line 55: change “contact with person” to “contact with people”.  Line 65: change “in the large teaching hospital” to “in a large teaching hospital  Line 78: change “the common psychological” to “common psychological”  Line 103-104: we removed this sentence.  Line 159-160: we added this sentence in the limitation (refer line 359-363).  Line 178: change “The malay” to “The Malay”  Line 282: change “The present study examines” to “The present study examined”  Line 285: change “in the government hospital” to “in the governments hospital”  Line 293: change “Woon LSC et al (2020) suggested” to “A study by Woon et al (2020) suggested”  Line 298: change “the risk to be infected” to “the risk of being infected”  Line 315: change “depression with being young HCWs” to “depression and being a young HCWs”  Line 338-3340: We revised the sentence “This present study showed a strong association between stress and feeling worried worry to get infected with COVID-19 among the staff. As a result, stress may suppress individual's confidence in treating critical patients.”  Line 374: We changed the “documenting” to “revealed”. |
